# Identification of Fungal Isolates in Patients With Pulmonary Tuberculosis Treated at a Tertiary Care Hospital

**DOI:** 10.7759/cureus.37664

**Published:** 2023-04-16

**Authors:** Sweta Muni, Kamlesh Rajpal, Rakesh Kumar, Ritu Kumari, Richa Sinha, Shailesh Kumar, Namrata Kumari

**Affiliations:** 1 Microbiology, Indira Gandhi Institute of Medical Sciences, Patna, IND; 2 Microbiology, Sanjay Gandhi Institute of Medical Sciences, Lucknow, IND

**Keywords:** pulmonary tuberculosis, fungal pathogens, diagnosis, coinfection, candida

## Abstract

Introduction

Pulmonary tuberculosis (TB) has long been associated with opportunistic fungal infections and could prove lethal if these fungal infections are not detected in the early stages of tuberculosis. TB patients are mostly immunocompromised, and an association with a fungal infection fuels each other, thus decreasing host immunity and making it difficult to treat. Extensive use of antibiotics and steroids has resulted in increasing trends of these fungal infections globally.

Material and methods

This observational, retrospective hospital-based medical record review study was conducted in the Department of Microbiology at the Indira Gandhi Institute of Medical Sciences (IGIMS), Patna, Bihar, India. A total of 200 medical records of pulmonary tuberculosis patients diagnosed by using sputum as clinical samples were evaluated and analysed for two years, from January 2020 to December 2021. This study was started after approval from the institutional ethical committee. Data were obtained from the mycology test records from the Department of Microbiology and from the data files of the medical records section over a period of two years.

Results

Our study included the medical records of 200 pulmonary tuberculosis patients who underwent treatment at IGIMS Patna. Out of 200 patient records, 124 (62%) were males and 76 (38%) were females. The male-to-female ratio was 1.6:1. After analysis and evaluation of 200 medical records of pulmonary tuberculosis patients, fungal species were detected in 16 (8%) sputum samples. Among 16 culture-positive sputum samples, 10 (8.06%) and six (7.1%) were diagnosed in male and female patients, respectively. Fisher's exact test showed a non-significant two-sided p-value of 1.000 with a relative risk of 0.9982. The prevalence, or positivity rate, was 8% in two years. The age group of 31-45 years had the most fungal co-infection at 37.5%. Among the fungal isolates, 5/16 (31.25%) were yeasts, and the remaining 11/16 (68.75%) isolates were mycelial fungi.

Conclusion

According to the findings of the present study, pulmonary fungal infections co-exist in tuberculosis patients, although the prevalence rates of all the coinfections are low and statistically not significant. Being chronic in nature and with confusing clinical and radiological findings, these fungal infections are misdiagnosed as reactivation of tuberculosis. Hence, the increasing rate of morbidity and mortality can decrease if adequate measures are taken for the diagnosis at an early stage and appropriate treatment of these fungal mycoses with antifungal therapy is instituted.

## Introduction

There has been a recent surge in cases of fungal infections in the past few decades, posing a diagnostic challenge. The true burden of these fungal infections still remains underdiagnosed [1]. Many factors are implicated in the increase in fungal infection, but the most common causes remain pulmonary tuberculosis (PTB), HIV/AIDS, and extensive use of immunosuppressive drugs [2]. Extensive use of antibiotics and steroids has also resulted in increasing trends of these fungal infections globally.

Tuberculosis is a potentially serious chronic lung disease that is caused by Mycobacterium tuberculosis and characterised by injury to the lung parenchyma with a granulomatous lesion along with epithelioid macrophages and Langhans giant cells with fibroblasts and collagenous tissue, as well as hallmark caseous necrosis in the centre [3]. Patients harbouring these pulmonary fungal infections have increased in recent times, and opportunistic fungal infections are mainly implicated in these cases [4].

The fungi and their spores colonise the lung parenchyma by various modes, such as inhalation, blood route, or by reactivation of a dormant infection. There is an ample number of fungi and their spores in the environment, and they pose a major challenge in patients with tuberculosis of the lungs [5]. There have been studies that suggest that the chronic nature of tuberculosis tends to be more lethal when it is associated with annexation by parasitic fungi, as not only does it make the tuberculosis infection more virulent, but it also adds to various complications and even death [6].

In a developing nation like India, the treatment of tuberculosis mostly remains empirical. Presumptive or undiagnosed cases of pulmonary tuberculosis without anti-tubercular therapy (ATT) or diagnosed or active cases on ATT will determine the extent of potential fungal pathogenic infections and their spectrum. Lack of definitive diagnosis and prompt treatment of pulmonary tuberculosis leads to further immunocompromised conditions and increases the chances of mycotic infection. Lack of correlation between fungal pathogens and pulmonary tuberculosis cases leads to poor clinical response to ATT treatment because the patients are receiving only anti-tubercular drugs without any anti-fungal drugs. The addition of antifungal drugs will prevent further progression of the disease, giving way to better outcomes and recovery [7].

TB patients, especially pulmonary cases, have long been associated with various Candida species [8]. Those patients who are on immunosuppressive drugs or have previously been treated with them generally harbour candida. Also, invasive candidiasis can occur in an immunocompetent individual as TB infection impairs the host's immunity, thus increasing the risk of pathogenic fungal infection [9]. Studies done by various researchers showed the presence of Aspergillus species like niger and fumigatus, Histoplasma capsulatum, and Cryptococcus neoformans as the major fungal pathogens in patients with pulmonary TB [10].

The presence of fungal pathogens in cases of pulmonary TB adds to the chronicity of the disease. This study was done to find out the division and percentage of probable fungal isolates that have an association with pulmonary TB infection. Also, it was sought to isolate and phenotypically characterise pulmonary mycotic agents from patients with pulmonary tuberculosis at the Indira Gandhi Institute of Medical Sciences (IGIMS) in Patna, India.

## Materials and methods

This was an observational, retrospective hospital-based medical record review study conducted in the microbiology department of IGIMS, Patna, Bihar, India. A total of 200 medical records of pulmonary tuberculosis patients diagnosed by using sputum as clinical samples were evaluated and analysed for two years, from January 2020 to December 2021. This study was started after approval from the Institutional Ethical Committee [449/IEC/IGIMS/2022]. Data from pulmonary tuberculosis tests and mycology tests were obtained from the records of the microbiology department and from the data files of the medical records section.

Inclusion Criteria

Medical records of patients included for analysis were: (1) patients of all age groups and all genders; (2) patients with clinical symptoms of pulmonary tuberculosis irrespective of HIV status; (3) patients with chronic conditions like diabetes mellitus, hypertension, and chronic obstructive pulmonary diseases; and (4) patients with a history of smoking or alcohol use.

Exclusion Criteria

Medical records of patients excluded from analysis were: (1) healthy subjects without features of pulmonary tuberculosis and multidrug-resistant TB cases; (2) patients with miliary tuberculosis; and (3) patients with a history of immunosuppressive therapy or immunocompromised disease.

As per the protocol of the Department of Microbiology at IGIMS, Patna, Bihar, if a patient's sputum sample tests positive for tuberculosis, the same sample is sent for a mycology test, and the following process is followed for the processing of the clinical specimens, the diagnosis, and the identification of fungal isolates.

Processing of Clinical Specimens

Sputum samples were collected early in the morning from the study participants and stored in a leak-proof bottle with a wide opening for fungal analysis for two consecutive days.

Diagnosis of Fungal Isolates

Isolation and characterization of fungi: inoculation of unprocessed sputum was done directly on Sabouraud’s dextrose agar (SDA) and Brain Heart Infusion agar tubes, respectively, supplemented with chloramphenicol. The transportation of inoculated tubes to the laboratory was done under aseptic precautions. The two tubes were incubated at 25°C and 37°C, respectively, under aerobic conditions for 28 days. A routine examination of the culture plates was done to rule out any fungal growth at least three times a week.

Identification

Mould: Identification of mycelia was based on their microscopic and macroscopic features. For microscopic features of mycelial fungi, staining with lactophenol cotton blue (LPCB) was done. A clean glass slide was taken, and a drop of LPCB stain was put on it. For the staining process, a section of fungal culture was taken and put on clean glass slides with LPCB. A cover slide was put on the stained preparation, and then, using 10x and 40x magnifications of the microscope, characteristics such as conidia, chlamydospores, reproductive structure morphology, and the nature of hyphae were noted. Macroscopically, the physical characteristics, texture, pigmentation, and rate of growth of cultures were seen.

Yeast: Using CHROMagar Candida (CHROMagar Candida, France), and as per the protocol of the manufacturer, the yeasts were recognised by following standard testing protocols.

Statistical Analysis

The collected data were entered into a Microsoft Excel sheet for result interpretation. The analysis of this was done using the Statistical Package for the Social Sciences (SPSS) software (IBM, California, USA), version 20.0. Period prevalence rate, Fisher's exact test, and relative risk were used to determine the degree of association between two categorical variables, and values of p ≤ 0.05 at 95% confidence intervals were considered statistically significant.

## Results

Patient characteristics in fungi distribution

Our study included the medical records of 200 pulmonary tuberculosis patients who underwent treatment at IGIMS, Patna. Out of 200 patient records, 124 (62%) were males and 76 (38%) were females. The male-to-female ratio was 1.6:1. After analysis and evaluation of 200 medical records of pulmonary tuberculosis patients, fungal species were detected in 16 (8%) sputum samples. Among 16 culture-positive sputum samples, 10 (8.06%) and six (7.1%) were diagnosed in male and female patients, respectively. The isolation rate of fungi was comparatively higher in males than in females. Statistically, no significant association was found between the prevalence of the pulmonary fungal isolation rate and the gender of patients (Table 1).

**Table 1:** The prevalence of fungal coinfection in males and females Fisher's exact test showed a two-sided p-value of 1.000, considered not significant with a relative risk (RR) of 0.9982.

Gender	Number	Positive	Percentage (%)
Male	124	10	8.06
Female	76	6	7.1
Total	200	16	8

The age of the patients included all with a mean age of 41 years. The study subjects were divided into five categories based on age: ages 1-15, 16-30, 31-45, 46-60 and more than 60 years. In this study, the age group of 31-45 years had the highest rate of coinfection at 37.5%. In general, the period prevalence or positivity rate was higher in patients in the age group of 31-45. It depicted that middle-aged groups were slightly more affected than younger or older groups (Table 2).

**Table 2:** Prevalence of fungal coinfection in TB patients according to different age groups Period prevalence (for two years) or positivity rate was 8% for all patients. Period prevalence or positivity rates were 4%, 9.4%, 11.8%, 5%, and 9.4% in age groups of 1-15, 16-30, 31-45, 46-60 and >60 years, respectively.

Age group	Number of patients (n=200)	Fungal coinfection n=16 (%)
1-15	25	1 (6.25%)
16-30	32	3(18.75%)
31-45	51	6(37.5%)
46-60	60	3(18.75%)
>60	32	3(18.75%)

Division of fungal isolates in TB patients

Our study shows that out of a total of 200 sputum-positive TB samples, the pathogenic fungus was isolated from 16 patient samples. Among the fungal isolates, 5/16 (31.25%) were yeasts containing Candida albicans (3/16; 18.75%) and non-Candida albicans (2/16; 12.5%). The remaining 11/16 (68.75%) isolates were mycelial fungi, where Aspergillus spp. (8/16; 50%), Penicillium spp. (2/16; 12.5%), and Mucor spp. (1/16; 6.25%) were isolated (Table 3).

**Table 3:** Distribution of fungal isolates

Fungal species (n=16)	Frequency	Percentage (%)
Candida albicans	3	18.75
Candida tropicalis	1	6.25
Candida krusei	1	6.25
Aspergillus flavus	3	18.75
Aspergillus fumigatus	4	25
Aspergillus niger	1	6.25
Mucor racemosus	1	6.25
Penicillium species	2	12.5

Sociodemographic profiles of patients were also studied according to their body mass index, smoking and drinking habits, chronic medical conditions, and associated fungal infection (Table 4).

**Table 4:** Association of TB and fungal coinfection with the demography of the study group Chronic conditions: diabetes mellitus, hypertension, and chronic obstructive pulmonary disease

Sociodemographic variable		Tuberculosis patients (n = 200)	Tuberculosis- Fungal coinfection (n =16)
BMI	<18.5	140 ( 70%)	10 (62.5%)
	>18.5	60 (30%)	6 (37.5 %)
Smoking	Yes	120 (60%)	9 (56%)
	No	80 (40%)	7 (44%)
Drinking	Yes	75 (37.5%)	4 (25%)
	No	125 (62.5%)	12 (75%)
Chronic conditions	Yes	95 (47.5%)	13 (81%)
	No	105 (52.5%)	3 (19%)

## Discussion

Tuberculosis is considered a serious health problem as well as a difficult disease to treat that occurs as a global problem with a varying incidence and prevalence rate. An increase in the prevalence rate of opportunistic fungal infections, which actually do not become pathogenic in normal, healthy individuals, has been documented in recent times. These generally become pathogenic in persons who have compromised immunity due to underlying diseases, increased consumption of broad-spectrum antibiotics, or who harbour pulmonary tuberculosis. The fungal coinfection adds to the morbidity of pulmonary tuberculosis cases and becomes difficult to treat.

The present study analysed sputum samples from 200 TB patients and studied coinfections with fungi in them. According to the present study, males were more affected by pulmonary TB, and their rates of coinfection with fungi were higher than those of females. The reason for this increased exposure to the external environment and surroundings in males is that more males work outside, the incidence of smoking is higher in them, and also men have greater access to healthcare facilities in developing countries [11]. Pulmonary fungal infection with Aspergillus occurs most commonly in individuals in the middle age group as compared to elderly people and is reported to be more common in male patients [12,13].

The result of our study was consistent with the findings of other researchers like Kosmidis C et al. [12] and Kohno S et al. [13], as the incidence of fungal mycosis was higher in the age group of 35 years and older than in much younger individuals, with males having more cases among them than their female counterparts. Various studies, however, concluded that people in their third and fourth decades were mostly associated with these fungal infections [14,15].

This finding also suggests that age is dependent on these mycotic infections, as lower ages below 10 years did not show the presence of the systemic infection, and ages greater than 60 years also showed very low prevalence, with mostly middle-aged patients showing high prevalence, possibly due to the high environmental exposure of this age group, particularly those having secondary infections. Aspergillus species like fumigatus, flavus, and niger, along with Candida albicans, were the most commonly diagnosed isolates from sputum in TB cases in the present study. This result is supported by a study done by Osman NM et al., where, in 50 TB cases, the most common fungal isolate was A. fumigatus among Aspergillus species [16].

Many other studies that have been done worldwide have documented Aspergillus species as the major fungus in patients supposed to be affected by tuberculosis of the lungs [17-18]. There was some variation in the Aspergillus species isolated, as the predominant species was A. fumigatus detected from a sputum sample in the present study, while a study done by Razmpa et al. detected a higher number of A. flavus [19].

In the present study, Candida albicans was the most frequent isolate among the yeast group, as recovered from 18.75% of patients, followed by Candida tropicalis (6.25%) and Candida krusei (6.25%), which is contrary to the study done by other researchers, who reported higher percentages of Candida albicans as 50% and 62% in patients, respectively [20,21]. In one of the studies done around a similar place as the present study over a one-year period, 75 samples of sputum suspected of pulmonary TB showed that Candida albicans was reported in 44.4% of cases, followed by A. niger in 33.3% of cases [22]. In contrast to these findings, our study showed the Aspergillus species as the most important species of fungi.

Numerous studies on cases of fungal infection have shown the harbouring of lung parenchyma with moulds like Aspergillus in some patients who are diagnosed with chronic obstructive pulmonary disease, lung abscess, cystic fibrosis of the lungs, and TB and have been associated with a high number of deaths and an improper prognosis and course of the diseases [23]. Various other researchers all over the world have also done similar studies recently, which have also shown comparable results with the present study (Table 5).

**Table 5:** Studies by different authors on TB-fungus coinfection

Study	Year	Country	TB-fungus coinfection/total sample	Yeast prevalence	Mould prevalence (%)
Bajare B et al. [24]	2019	India	33/171 (20%)	Candida species (48.4%)	42%
Hussein HM et al. [25]	2021	Iraq	2/30 (6.6%)	--	--
Yan et al. [26]	2023	China	145/1151(12%)	--	--
Bitew A et al. [2]	2021	Ethiopia	127/163 (78%)	Candida species (80%)	20%
Sani FM et al. [27]	2020	Nigeria	13/216 (6%)	Candida species (27%)	73%
Amri MRJ et al. [28]	2017	Iran	16/130 (12%)	Candida species (37.5%)	62.5%

Limitations

The present study was done in a single centre with limited sample size and limited access to data. The authors recommend multicentric studies with a larger sample size to achieve better results.

## Conclusions

Pulmonary fungal infections co-exist in tuberculosis patients, according to the findings of the present study, although the prevalence rates of all the coinfections were low and statistically not significant. The presence of these infectious agents in TB patients poses a great risk of making the patients more ravaged due to the pathogenic synergism existing among the infections. Being chronic in nature and with confusing clinical and radiological findings, these fungal infections are misdiagnosed as reactivation of tuberculosis. Hence, the increasing rate of morbidity and mortality can decrease if adequate measures are taken for the diagnosis at an early stage and appropriate treatment of these fungal mycoses.
